# Repetitive Complete Molar Pregnancy in a 54-Year-Old Patient in a Time Distance of Eighteen Years from the First Incident: Case Report and Mini Review

**DOI:** 10.1155/2011/351267

**Published:** 2011-10-26

**Authors:** Polychronis Oikonomidis, Basileios Pergialiotis, Elina Pitsouni, Stavros Natsis, Antonios Lagkadas, Konstantinos Giannakopoulos

**Affiliations:** Department of Gynecology, General Hospital of Athens “Laiko”, Ag. Thoma 17, Goudi, 15232 Athens, Greece

## Abstract

We report a case of complete hydatidiform mole in a 54-year-old patient referred to Gynecology department of General Hospital of Athens “Laiko”, with history of previous molar pregnancy at the age of thirty-six. Our purpose was to indicate the advanced maternal age beside the long recurrence distance of the disease, which was eighteen years after the first molar pregnancy. Our diagnostic approach was through measurement of serum beta-human chorionic gonadotropin (**β**-HCG) and pelvic ultrasound evaluation, and the chosen therapeutic approach was abdominal hysterectomy and bilateral salpingoophorectomy.

## 1. Introduction

Hydatidiform moles (HMs) essentially represent an abnormality of placental development as a consequence of overexpression of paternally derived genes and are therefore examples of abnormalities of imprinting. They are associated with structural placental abnormalities and fetal developmental defects and are characterized pathologically from nonmolar pregnancies by the presence of abnormal trophoblast hyperplasia. They are simply divided into two major subtypes, complete (CHM) and partial (PHM) hydatidiform mole, according to pathologic and genetic features [1–3].

Studies from the United States and England have found that women with a history of one molar pregnancy (partial, complete, or persistent GTN) have an approximately 1% chance of recurrence in subsequent pregnancy (compared to a 0.1% incidence in the general population of the United States) [4–6]. The recurrence rate is much higher after two molar pregnancies (16 to 18%) [7–10]. 

## 2. Description of Case

A 54-year-old patient, gravida 3, para 2, was referred to General Hospital of Athens, “Laiko” with intermittent, irregular vaginal bleeding for the last three months and a concomitant, mild abdominal pain.

The patient is a mother of two children, full-term cesarean deliveries. Last delivery was twenty years ago (at the age of 34). Two years later (age 36), she developed a complete molar pregnancy which was, at that time, managed with evacuation of the uterine contents by suction and curettage. 

The patient arrived in our department complaining from irregular menstrual period in the last year, with an interval of three months of amenorrhea. She had never used any contraceptive method and was average in weight, of medium socioeconomic status, and with no history of hypertension, diabetes, or symptoms of thyroid disease.

A thorough clinical history was taken. Physical examination, blood pressure, chest rays, and electrocardiogram were normal, abdominal and pelvic examination revealed enlargement of the uterus reaching about 17-week gestation, and speculum and bimanual examination demonstrated a healthy cervix with slight uterine bleeding.

**Figure 1:**
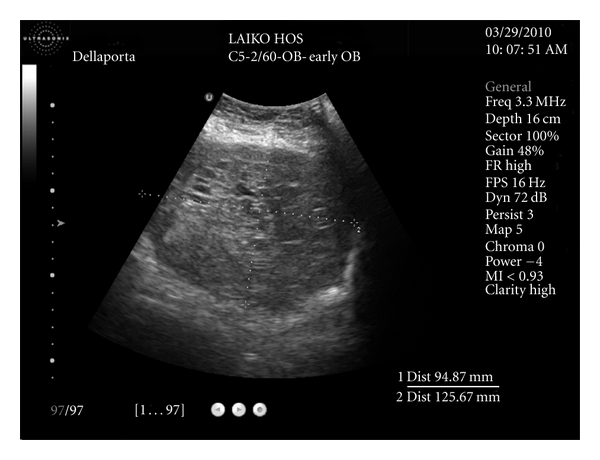
Explanatory legend: Transvaginal ultrasound of the patient. Snowstorm-like appearance. Uterus dimension: 94,87 mm × 125,67 mm.

Ultrasound evaluation showed a uterine cavity filled with central heterogeneous mass with anechoic spaces of varying size and shape. Moreover a subserous fibroid was found at the fundus with a size of 2 × 3 cm (Figure 1).

Maternal serum beta-chorionic gonadotrophin (*β*-HCG) levels were 97000 IU/L. She was admitted to hospital for preoperative assessment. Blood group and Rh factor, matching of two units of compatible blood, were prepared.

Differential diagnosis included (apart molar pregnancy and choriocarcinoma) germ cell tumors and other malignancy involving organs like the ovary, bladder, uterus, lung, liver, pancreas and stomach neoplasms, although *β*-HCG's relatively high levels turn away from these [11]. Considering the patient's age and with her concordant opinion, a laparotomy and hysterectomy with bilateral salpingo-oophorectomy (H.B.S.O.) were decided. 

Histopathology showed a complete mole with no evidence of invasion, and chromosomal analysis revealed paternal origin (46,XX).

Followup after two and four weeks of operation with serum *β*-HCG measurements showed that the level returned back to normal. The patient was followed for the next six months with *β*-HCG which did not show any abnormality.

## 3. Discussion

Hydatidiform mole is a gestational trophoblastic disorder due to abnormal gametogenesis and fertilization. There are two types of hydatidiform mole, namely, complete and partial. Its incidence increases at the extremes of reproductive age. Teenagers and perimenauposal women are most at risk [12]. Characteristically, women older than 35 years of age have a relative risk of 2.0, and women over 40 years of age have a 5- to 10-fold increase [13]. However a single study states that repetition of the disease (second molar pregnancy) seems to present within 2 to 4 years after the first incident (patients in the study were followed up for one year if partial mole was the diagnosis and for 2 years if complete mole was observed) [5]. Case reports in extreme cases of patients with multiple recurrences of molar pregnancies confirm these findings [14, 15].

Complete hydatidiform mole (CHM) results from the fertilization of an egg, from which the nuclear material has been lost or inactivated by a single sperm having 23,XX chromosomes which duplicates to 46,XX. This makes complete mole homozygous, female, and androgenic in origin [16]. Less frequently, fertilization occurs with two sperms resulting in either 46xx or 46xy heterozygous chromosomal constitution [17]. Biparental CHMs are reported in a limited number of studies and seem to be extremely rare [18, 19]. However, a study by Moglabey et al. in which several sisters had one or more CHM, it was found that all of them were biparental in origin, implying that familial repetitive HM is of biparental origin.

In partial hydatidiform mole, maternal chromosomes are present and the condition arises by diandry (one maternal and two paternal sets of chromosomes) [20]. 

The genetic background of mole is a matter of investigation in several studies. Genes implicated in familial forms and repetitive case reports are the *NLRP7* and *C6orf221* [21, 22]. Researchers found that *NLRP7* mutations at 19q13.4 were responsible for an increase in stochastic and mosaic aneuplodies during early embryo development [23]. Eventually, the resulting embryos survived through implantation or regressed and diseased, depending on their severity. The pathogenetic pathway between mutations of the *NLRP7* gene and the development of molar pregnancy is explained through an inability to express inflammatory response to various antigens and stimuli [23]. The *NLRP* genes (*Nucleotide-binding oligomerization domain, Leucine rich Repeat, and Pyrin domain containing Proteins*) seem to be interrelated with the mammalian reproductive system [24]. Further investigation of *NLRP7'*s structural polymorphisms may highlight its participation in the pathogenesis of hydatiform mole, rendering it a possible prenatal marker in the future.

Both forms of moles are potentially malignant. The risk of gestational trophoblastic neoplasia for partial mole is <5–10% and that of complete mole is 20%. The risk of recurrence of hydatidiform mole is 0.5–2.8% with a subsequent greater risk of developing invasive mole or choriocarcinoma [10]. The risk of repeat hydatidiform mole in next pregnancy is 1 : 76, while the risk after two past hydatidiform mole is 1 : 6.5 [6].

Molar pregnancy is an uncommon cause of abdominal pain and vaginal bleeding. In a retrospective case study, the authors stated abdominal pain with enlarged uterine size in 54% of women with mole and vaginal bleeding in another 75%.

In patients with a second molar pregnancy, the pathological type is not necessarily of the same type. Of women with a complete molar pregnancy who have another molar pregnancy, 81% had another CHM, whilst 19% had a PHM with their next molar pregnancy. Similarly, of those who had a PHM for their first molar pregnancy and then went on to have another molar pregnancy, 68% had another PHM and 32% a CHM [25].

Literature often reports recurrence or repetition of the disease, but this usually happens in a short period of time [26]. We could not detect in literature cases that recurred after 5 years of first disease, so we consider that the case we report is fairly rare and interesting, since the recurrence occurred 18 years after the first event. 

## 4. Conclusion

Complete hydatiform mole is a disease that may recur at any timeline after the first incident. Although most cases are described as having short intervals of time and usually of the same histological type, this is not an unbreakable rule. Women in the 6th decade of life usually present with vaginal bleeding as a result of menstrual abnormalities resulting from induction to menopause. Endometrial polyps, endometrial hyperplasia, and endometrial cancer are other possible causes of vaginal bleeding. However, when levels of *β*-HCG are high, molar pregnancy is the most possible diagnosis, as its incidence increases at ages <20 years and >40 years. 

## Abbreviations

HM: Hydatidiform mole
CHM: Complete hydatidiform mole
PHM: Partial hydatidiform mole
GTN: Gestational trophoblastic neoplasm
*β*-hCG: Beta-human chorionic gonadotropin
Rh: Rhesus
HBSO: Hysterectomy with bilateral salpingo-oophorectomy.

## References

[1] Callen PW (2008). *Ultrasonography in Obstetrics and Gynecology*.

[2] Berkowitz R (2003). *Gestational Trophoblastic Disease*.

[3] Altieri A, Franceschi S, Ferlay J, Smith J, La Vecchia C (2003). Epidemiology and aetiology of gestational trophoblastic diseases. *Lancet Oncology*.

[4] Berkowitz RS, Tuncer ZS, Bernstein MR, Goldstein DP (2000). Management of gestational trophoblastic diseases: subsequent pregnancy experience. *Seminars in Oncology*.

[5] Lorigan PC, Sharma S, Bright N, Coleman RE, Hancock BW (2000). Characteristics of women with recurrent molar pregnancies. *Gynecologic Oncology*.

[6] Bagshawe KD, Dent J, Webb J (1986). Hydatidiform mole in England and Wales 1973–83. *The Lancet*.

[7] Altman AD, Bentley B, Murray S, Bentley JR (2008). Maternal age-related rates of gestational trophoblastic disease. *Obstetrics and Gynecology*.

[8] Tsukamoto N, Iwasaka T, Kashimura Y, Uchino H, Kashimura M, Matsuyama T (1985). Gestational trophoblastic disease in women aged 50 or more. *Gynecologic Oncology*.

[9] Berkowitz RS, Im SS, Bernstein MR, Goldstein DP (1998). Gestational trophoblastic disease. Subsequent pregnancy outcome, including repeat molar pregnancy. *Journal of Reproductive Medicine*.

[10] Sand PK, Lurain JR, Brewer JI (1984). Repeat gestational trophoblastic disease. *Obstetrics and Gynecology*.

[11] Olsen TG, Barnes AA, King JA (2007). Elevated hCG outside of pregnancy—diagnostic considerations and laboratory evaluation. *Obstetrical and Gynecological Survey*.

[12] Bandy LC, Clarke-Pearson DL, Hammond CB (1984). Malignant potential of gestational trophoblastic disease at the extreme ages of reproductive life. *Obstetrics and Gynecology*.

[13] Palmer JR (1994). Advances in the epidemiology of gestational trophoblastic disease. *Journal of Reproductive Medicine*.

[14] Koc S, Ozdegirmenci O, Tulunay G, Ozgul N, Kose MF, Bulbul D (2006). Recurrent partial hydatidiform mole: a report of a patient with three consecutive molar pregnancies. *International Journal of Gynecological Cancer*.

[15] Sahraoui W, Hajji S, Haouas N (2006). Recurrent hydatidiform mole. Case report of 9 successive molar pregnancies. *Tunisie Medicale*.

[16] Wolf NG, Lage JM (1995). Genetic analysis of gestational trophoblastic disease: a review. *Seminars in Oncology*.

[17] Lawler SD, Fisher RA, Dent J (1991). A prospective genetic study of complete and partial hydatidiform moles. *American Journal of Obstetrics and Gynecology*.

[18] van der Smagt JJ, Scheenjes E, Kremer JAM, Hennekam FAM, Fisher RA (2006). Heterogeneity in the origin of recurrent complete hydatidiform moles: not all women with multiple molar pregnancies have biparental moles. *BJOG: An International Journal of Obstetrics and Gynaecology*.

[19] Helwani MN, Seoud M, Zahed L, Zaatari G, Khalil A, Slim R (1999). A familial case of recurrent hydatidiform molar pregnancies with biparental genomic contribution. *Human Genetics*.

[20] Lawler SD, Fisher RA, Pickthall VJ (1982). Genetic studies on hydatidiform moles. I. The origin of partial moles. *Cancer Genetics and Cytogenetics*.

[21] Parry DA, Logan CV, Hayward BE (2011). Mutations causing familial biparental hydatidiform mole implicate c6orf221 as a possible regulator of genomic imprinting in the human oocyte. *The American Journal of Human Genetics*.

[22] Wang CM, Dixon PH, Decordova S (2009). Identification of 13 novel NLRP7 mutations in 20 families with recurrent hydatidiform mole; missense mutations cluster in the leucine-rich region. *Journal of Medical Genetics*.

[23] Deveault C, Qian JH, Chebaro W (2009). NLRP7 mutations in women with diploid androgenetic and triploid moles: a proposed mechanism for mole formation. *Human Molecular Genetics*.

[24] Tian X, Pascal G, Monget P (2009). Evolution and functional divergence of NLRP genes in mammalian reproductive systems. *BMC Evolutionary Biology*.

[25] Sebire NJ, Fisher RA, Foskett M, Rees H, Seckl MJ, Newlands ES (2003). Risk of recurrent hydatidiform mole and subsequent pregnancy outcome following complete or partial hydatidiform molar pregnancy. *BJOG: An International Journal of Obstetrics and Gynaecology*.

[26] Di Cintio E, Parazzini F, Rosa C, Chatenoud L, Benzi G (1997). The epidemiology of gestational trophoblastic disease. *General and Diagnostic Pathology*.

